# The current status of methods used by the elderly for suicides in England and Wales

**DOI:** 10.5249/jivr.v3i2.78

**Published:** 2011-07

**Authors:** Ajit Shah, Laura Buckley

**Affiliations:** ^*a*^International School for Communities, Rights and Inclusion, University of Central Lancashire, Preston, United Kingdom and Consultant Psychiatrist, West London Mental Health NHS Trust, London, United Kingdom.; ^*b*^Student Intern, International School for Communities, Rights and Inclusion, University of Central Lancashire, Preston, United Kingdom.

## Abstract

**Background:**

Suicide rates in older people in England and Wales have declined in recent years. The challenge, therefore, is to sustain this downward trend. Better understanding of the current methods of suicide used by older people may inform strategies to sustain this decline.

**Methods:**

A study to ascertain the up to date status of methods of suicides used by older people in England and Wales was undertaken using the latest available national mortality data (suicides and open verdicts) from the Office of National Statistics for the years 2001 to 2007. Suicide in this study is measured by and includes the combination of deaths due to suicides (ICD-10 codes X60-X84) and open verdicts (ICD-10 codes Y10-Y34). The chi square test (corrected for continuity) was used to examine the differences in the methods of suicide between: (i) those under the age of 65 years and those aged 65 years and over in both sexes; (ii) males and females aged 65 years and over.

**Results:**

Hanging, strangulation and suffocation (males, 40.2%; females 20.1%), drowning and submersion (males, 8.2%; females 11.4%), and other and unspecified drugs, medicaments and biological substances (males, 8%; females, 20.4%) were the most common methods of suicide in older people. There were significant differences in the methods of suicide used by older men and women, and by older and younger people in both sexes. Suicide by unspecified means was common in both older men and women and higher in older women than older men. Unfortunately, data on the potential method of suicide is not available for these "unspecified means.

**Conclusions:**

The clear differences in the methods of suicide between older and younger people and between older men and women suggest a need to develop different strategies to reduce access to these methods of suicide for different age and sex groups. Also, potentially preventable methods of suicide may be hidden behind suicide by unspecified means. Therefore, there is a need to accurately ascertain and document methods of suicide, as they may offer opportunities for prevention.

## Introduction

Suicide rates in older people have declined in both sexes over the 12-year period (1985-1996) and 24-year period (1979-2002) in England and Wales^1,2^and in United Kingdom (UK) in general^3,4^respectively. For example, in England and Wales the decline between 1979 and 2002 was as follows: in males 65+-74 years from 16.3 to 8.6 per 100,000 population; in males 75+ from 24.9 to 10.6 per 100,000 population; in females 65+-74 years from 12.0 to 3.1 per 100,000 population; and in females 75+ from 8.9 to 4.4 per 100,000 population.^4^This decline was associated with increased prescribing of antidepressants in the category of selective serotonin reuptake inhibitors,^5^and with measures of improved healthcare for the elderly^6,7^including an increase in the number of general practitioners, hospital medical staff, out-patient appointments for mental illness, and field social workers and day centre staff.^7^Other possible explanations included:^1,3,4^legislation requiring general practitioners to offer annual physical and mental examination to those aged over 75 years; the Defeat Depression Campaign organized by the Royal College of Psychiatrist; the National Confidential Enquiry into Suicides and Homicides; the governmental “Our Healthier Nation” suicide reduction targets; the National Service Frameworks for Mental Health and for Older People; and the National Suicide Prevention Strategy. The challenge, therefore, will be to sustain this downward trend in suicide rates in older people.

The British government has targeted to reduce suicide rates in the general population by at least one-sixth of the 1996 baseline by 2010.^8^Reduction in suicide rates in older people may be an important contributor to this target because traditionally suicide rates increased with ageing.^9,10^Therefore, a better understanding of the methods used by older people for suicides may lead to the development of targeted preventative strategies to meet the challenge of sustaining this observed decline in suicide rates in older people over time in England and Wales.^11,12^

Methods used by older people for suicides in England and Wales have been critically examined using data from coroner’s courts,^13-18^psychological post-mortem studies,^19,20^and national mortality statistics.^1,2,21-24^Rates of suicide due to self-poisoning in England and Wales declined between 1974 and 1984^21,22^and between 1979 and 2001,^24^but remained constant between 1993 and 1999.^23^Moreover, rates of suicides in older people due to solid or liquid substances, hanging, strangulation and suffocation, drowning and submersion, firearms and explosives, and jumping from high place declined between 1985 and 1996 in England and Wales.^1^In addition, detoxification of domestic gas, restricting prescriptions for barbiturates and the introduction of catalytic converters in cars leading to a reduction in carbon monoxide poisoning, have all led to a decline in suicide rates.^11,12,21,24,25^

However, all these studies, except one,^25^used at least a decade old data to examine suicide rates and types. Therefore, a study to ascertain the current status of methods used by older people for suicides in England and Wales using the latest available national mortality data was undertaken.

## Methods

**Measures of suicides**

Data on suicides and open verdicts (pertaining to deaths from injury and poisoning), for the seven year period 2001 to 2007, was ascertained from the Office of National Statistics(http://www.statistics.gov.uk/statbase/Product.asp?Vlnk =618). The seven year study period 2001 to 2007 was chosen because the Office of National Statistics commenced using ICD-10 codes for suicides and open verdicts in 2001, and these data sets are the latest available using ICD-10 codes. Data on suicides and open verdicts were available for 21 five-year age-bands (age groups) for both sexes of less than 1 year, 1-4 years, 4-9 years, 10-14 years, 15-19 years, 20-24 years, 25-29 years, 30-34 years, 35-39 years, 40-44 years, 45-49 years, 50-54 years, 55-59 years, 60-64 years, 65-69 years, 70-74 years, 75-79 years, 80-84 years, 85-89 years, 90-94 years and 95+ years. This data was collapsed into the following age-bands for both sexes: those under the age of 65 years; and those aged 65 years and over.

Deaths due to suicides were defined by the ICD-10 categories of X60 to X84, referring to intentional self harm. Deaths with open verdicts were defined by the ICD-10 categories of Y10-Y34, referring to injury by undetermined intent. In England and Wales, the coroner can only return a verdict of suicide if death by suicide it can be proved beyond a reasonable doubt that the cause of death is suicide and not another cause. Therefore, some true suicides may be misclassified under an open verdict when suicides cannot be proved beyond a reasonable doubt. Thus, suicide in this study is measured by and includes the combination of deaths due to suicides (ICD-10 codes X60-X84) and open verdicts (ICD-10 codes Y10-Y34)^1,2,24,26^(see footnote in Table 1). There were no other inclusion or exclusion criteria.

**Table:** Table 1:**Suicides by age and sex**

ICD-10 Code	Males <65 N(%)	Males 65+N (%)	X2 (*)(P)	Females <65 N(%)	Females 65+ N (%)	X2 (**)(P)	Over 65 men vs women X2 (P)
X60 & Y10	343 (1.5)	121 (3.4)	P<0.001	281 (4.1)	111 (6.1)	P<0.001	P<0.001
X61 & Y11	1053 (4.5)	162 (4.6)	NS	786 (11.6)	205 (11.3)	NS	P<0.001
X62 & Y12	825 (3.6)	91 (2.6)	P=0.004	343 (5)	95 (5.2)	NS	P<0.001
X63 & Y13	40 (0.2)	7 (0.2)	NS	30 (0.01)	3 (0.2)	NS	NS
X64 & Y14	1405 (4.5)	283 (8.0)	P<0.001	1136 (16.7)	370 (20.4)	P<0.001	P<0.001
X65 & Y15	36 (0.2)	5 (0.1)	NS	8 (0.1)	1 (0.1)	NS	NS
X66 & Y16	18 (0.1)	3 (0.1)	NS	12 (0.2)	0 (0)	NS	NS
X67 & Y17	1413 (6.1)	145 (4.1)	P<0.001	147 (2.2)	14 (0.8)	P<0.001	P<0.001
X68 & Y18	14 (0.1)	2 (0.1)	NS	5 (0.7)	3 (0.2)	NS	NS
X69 & Y19	47 (0.2)	11 (0.3)	NS	17 (0.3)	5 (0.3)	NS	NS
X70 & Y20	11523 (49.6)	1418 (40.2)	P<0.001	2040 (30)	336 (20.1)	P<0.001	P<0.001
X71 & Y21	827 (3.6)	289 (8.2)	P<0.001	344 (5.1)	207 (11.4)	P<0.001	P<0.001
X72 & Y22	7 (0.03)	2 (0.1)	NS	0 (0)	1 (0.1)	NS	NS
X73 & Y23	265 (1.1)	86 (2.4)	P<0.001	17 (0.3)	0 (0)	NS	P<0.001
X74 & Y24	208 (0.9)	95 (2.7)	P<0.001	11 (0.2)	1 (0.1)	NS	P<0.001
X75 & Y25	4 (0.02)	1 (0.03)	NS	2 (0.01)	0 (0)	NS	NS
X76 & Y26	297 (1.3)	50 (1.4)	NS	152 (2.2)	20 (1.1)	P=0.003	NS
X77 & Y27	2 (0.01)	1 (0.03)	NS	0 (0)	1 (0.1)	NS	NS
X78 & Y28	555 (2.4)	145 (4.1)	P<0.001	79 (1.2)	30 (1.7)	NS	P<0.001
X79 & Y29	2 (0.01)	0 (0)	NS	0 (0)	1 (0.1)	NS	NS
X80 & Y30	659 (2.8)	102 (2.9)	NS	260 (3.8)	47 (2.6)	P=0.014	NS
X81 & Y31	848 (3.7)	88 (2.5)	P<0.001	220 (3.2)	31 (1.7)	P<0.001	NS
X82 & Y32	39 (0.2)	0 (0)	NS	13 (0.2)	0 (0)	NS	NS
X83 & Y33	1663 (7.0)	214 (6.1)	P=0.04	572 (8.1)	164 (9)	NS	P<0.001
X84 & Y34	1156 (5.0)	204 (5.8)	P=0.046	324 (4.8)	142 (7.8)	P<0.001	P=0.005
Total	23219 (100)	3525 (100)	6798 (100)	1818 (100)			

* = Men under 65 Vs Men 65 years and over. ** = Women under 65 Vs Women 65 years and over. NS= Not significant. X60 & Y10– Poisoning by and exposure to non-opioid analgesics, antipyretics and antirheumatics. X61 & Y11 - Poisoning by and exposure to anti-epileptic, sedative-hypnotic, antiparkinsonism and psychotropic drugs. X62 & Y12- Poisoning by and exposure to narcotics and psychodysleptics [hallucinogens]. X63 & Y13- Poisoning by and exposure to other drugs acting on the autonomic nervous system. X64 & Y14- Poisoning by and exposure to other and unspecified drugs, medicaments and biological substances. X65 & Y15–  Poisoning by and exposure to alcohol. X66 & Y16- Poisoning by and exposure to organic solvents and halogenated hydrocarbons and their vapours. X67 & Y17- Poisoning by and exposure to other gases and vapours. X68 & Y18- Poisoning by and exposure to pesticides. X69 & Y19- Poisoning by and exposure to other and unspecified chemicals and noxious substances. X70 & Y20– Harm by hanging, strangulation and suffocation. X71& Y21 – Self-harm by drowning and submersion. X72 & Y22- Self-harm by handgun discharge. X73 & Y23- Self-harm by rifle, shotgun and larger firearm discharge. X74 & Y24- Self-harm by other unspecified firearm discharge. X75 & Y25- Self-harm by explosive material. X76 & Y26 - Self-harm by smoke, fire and flames. X77 & Y27- Self-harm by steam, hot vapours and hot objects. X78 & Y28- Self-harm by sharp objects. X79 & Y29 - Self-harm by blunt objects. X80 & Y30- Self-harm by jumping from a high place. X81 & Y31- Self-harm by jumping or lying before moving object. X82 & Y32- Self-harm by crashing of motor vehicle. X83 & Y33- Self-harm by other specified means. X84 & Y34- Self-harm by other unspecified means

**Data analysis**

Descriptive statistics were used to describe the frequency of individual methods used for suicide. The number of suicides with the different methods of suicide for those under the age of 65 years and those aged 65 years and over, in both sexes, were calculated for the entire seven-year study period. The chi square test (corrected for continuity) was used to examine the differences in the methods of suicide between: (i) those under the age of 65 years and those aged 65 years and over in both sexes; (ii) males and females aged 65 years and over.

## Results

**The number of suicides**

There were a total of 35360 (true suicide, 22750; open verdict, 12610) suicides during the seven-year study period: 23219 (65.7%) (true suicide, 14891; open verdict, 8328) in males under the age of 65 years; 3525 (9.9%) (true suicide, 2571; open verdict, 954) in males aged 65 years and over; 6798 (19.2%) (true suicide, 4096; open verdict, 2702) in females under the age of 65 years; and, 1818 (5.1%) (true suicide, 1192; open verdict, 626) in females aged 65 years and over.

**Methods of suicide**

Table 1illustrates the number of suicides among those under the age of 65 years and those aged 65 years and over, in both sexes, for each method of suicide.  The most common methods of suicide in order of frequency in men aged 65 years and over were: hanging, strangulation and suffocation (40.2%); drowning and submersion (8.2%); other and unspecified drugs, medicaments and biological substances (8%), and other unspecified means (5.8%). The most common methods of suicide in order of frequency in women aged 65 years and over were: other and unspecified drugs, medicaments and biological substances (20.4%), ; hanging, strangulation and suffocation (11.4%); drowning and submersion (20.1%); anti-epileptic, seda-tive-hypnotic, antiparkinsonism and psychotropic drugs (11.3%); and other unspecified means (7.8%).

**Methods of suicide in older men and women**

The following methods of suicide were significantly higher in older men than women: other gases and vapors (males, 4.1%; females, 0.8%; P<0.001; hanging, strangulation and suffocation (males, 40.2%; females,20.1; P<0.001; rifle, shotgun and larger firearm discharge (males, 2.4%; females, 0%; P<0.001; other unspecified firearm discharge; and sharp objects (males, 2.7%; females, 0.1%; P<0.001. The following methods of suicide were significantly higher in older women than men: non-opioid analgesics, antipyretics and antirheumatics (males, 3.4%; females, 6.1%; P<0.001); antiepileptic, sedative-hypnotic, antiparkinsonism and psychotropic drugs (males, 4.6%; females, 11.3%; P<0.001); narcotics and psychodysleptics [hallucinogens] (males, 2.6%; females, 5.2%; P<0.001; other and unspecified drugs, medicaments and biological substances (males 6.1%; females, 9%; P<0.001 ); drowning and submersion (males, 8.2%; females, 11.4% ; P<0.001; other specified means (males, 6.1%; females, 9%; P<0.001); and other unspecified means (males, 5.8% ; females, 7.8%; P=0.005).

**Methods of suicide in older and younger men and women**

The following methods of suicide were significantly higher in older men than younger men: non-opiod analgesics, antipyretics and antirheumatics (young, 1.5%; old, 3.4%; P<0.001; other and unspecified drugs, medicaments and biological substances (young, 4.5%; older, 8%; P<0.001; drowning and submersion (young, 3.6%; older, 8.2%; P<0.001; rifle, shotgun and larger firearms (young, 1.1%; older, 2,4%; P<0.001); other unspecified firearm discharge (younger, 0.9%; older, 2.7%; P<0.001; sharp objects (young, 2.4%; older, 4.1%; P<0.001); and, other unspecified means (young, 5%; older, 5.8%; P=0.046). The following methods were significantly less common in older men than younger men: narcotics and psychodysleptics [hallucinogens] (young, 3.6%; older, 2.6%; P=0.003); other gases and vapors (young, 6.1%; older, 4.1%; P<0.001; hanging, strangulation and suffocation (young, 49.6%; older, 40.2%; P<0.001); jumping or lying before moving objects (young, 3.7%; older, 2.5%; P<0.001; and other specified means (young, 7%; older, 6.1%; P=0.04).

The following methods of suicide were significantly higher in older women than younger women: non-opioid analgesics, antipyretics and antirheumatics (young, 4.1%; older, 6.1%; P<0.001); other and unspecified drugs, medicaments and biological substances (young, 16.7%; older, 20.4%; P<0.001); drowning and submersion (young, 5.1%; older, 11.4%; P<0.001); and, other unspecified means (young, 4.8%; older, 7.8%; P<0.001). The following methods of suicide were significantly lower in older women than younger women: other gases and vapors (younger, 2.2%; older, 0.8%; P<0.001); hanging, strangulation and suffocation (young, 30%; older, 20.1%; P<0.001); smoke, fire and flames (young, 2.2%; older, 1.1%; P=0.03); jumping from a high place (young, 3.8%; older, 2.6%; P=0.014); and jumping or lying before moving object (young, 3.2%; older, 1.7%; P<0.001).

## Discussion

Some methodological issues need consideration. First, national aggregate data may miss regional variations in suicide rates.^27^Second, some deaths with an open verdict may not have been suicides.^28^Third, the effect of age, period and cohort were not examined. The age of the suicide victim, the cohort the suicide victim belongs to and the time period of the study all can independently influence suicide rates.  Nevertheless, the data sets used for this study were the latest and most updated available from the Office of National Statistics.

Hanging,strangulation and suffocation,drowning and submersion,and other and unspecified drugs, medicaments and biological substances were the most common methods of suicide in older people and this has been reported in previous studies.^1,13-18,24^Another common method of suicide used by older people observed in the current study was other unspecified means, which has also been reported in previous studies.^16^Unfortunately, data on the potential method of suicide is not available for these “unspecified means”. Potentially preventable methods of suicide may be hidden behind the unspecified means of suicide. Therefore, there is a need to accurately ascertain and document methods of suicide as they may offer opportunities for prevention.

Violent methods, including hanging, strangulation and suffocation, rifle, shotgun and larger firearm discharge, other unspecified firearm discharge and sharp objects were more common in older men than women, and these have been reported in previous studies.^1,19,15-17,24^Poisoning by other gases and vapors was more common in older men than women in the current study, which is consistent with similar observations in England and Wales between 1985 and 1996,^1^and supports the observation that carbon monoxide poisoning was more common in older men than women in previous studies.^15,16^Self poisoning due to non-opioid analgesics, antipyretics and antirheumatics, anti-epileptic, sedative-hypnotics, antiparkinsonism and psychotropic drugs, narcotics and psychodysleptics, and other unspecified drugs were more common in older women than men, as has been observed in previous studies.^1,14-16^

Drowning and submersion were also more common in older women than men; a gender pattern which has been observed in a previous psychological autopsy study.^15^However, in contrast, drowning was more common in older men than women in England and Wales between 1985 and 1996.^1^Suicide by other and unspecified means was also more common in older women, but has not been reported previously. Again potentially preventable methods of suicide may be hidden behind unidentified methods of suicide.

Only one UK study has compared methods of suicide used by young victims and older victims in the last decade.^16^That study reported that drowning was more common and falling from a height less common in older men compared to younger men,^16^a finding which was also observed in the current study. A previous study reported that asphyxia was more common in older than younger women,^16^but the opposite was observed in the current study. However, many other differences in the methods of suicide between older people and younger people, in both sexes, were observed in the current study, and they have not been reported in the UK in the last decade.

There is unequivocal evidence that reducing access to methods of suicide, including detoxification of domestic gas, restricted prescribing of barbiturates and the introduction of catalytic converters in cars leading to a decrease in carbon monoxide poisoning, can reduce suicide rates and that this reduction can be sustained over time in previous studies.^11,12,21,24,25^There is, therefore, a need to focus on reducing the most prevalent methods of suicide in different age and sex groups, which could be achieved by developing strategies to reduce access to these most prevalent methods of suicide for different age and sex groups. Suicide by unspecified means is of particular importance because this method is common in both older men and women, higher in older women than men, and higher in older than younger women. Potentially preventable methods of suicide may be hidden behind unidentified methods of suicide. Therefore, there is a need to accurately ascertain and document the exact methods of suicide as they may offer opportunities for prevention.
